# Uncertainty work as ontological negotiation: adjudicating access to therapy in clinical psychology

**DOI:** 10.1111/1467-9566.13029

**Published:** 2019-11-26

**Authors:** Martyn Pickersgill

**Affiliations:** ^1^ Centre for Biomedicine, Self and Society Edinburgh Medical School University of Edinburgh Edinburgh UK

**Keywords:** access to therapy, clinical psychology, psychological therapy, mental health, ontology

## Abstract

Across the UK, wide‐ranging efforts have been made to enhance citizen access to psychological therapy. Clinical psychologists are key providers of and gatekeepers for therapy. This article is concerned with how clinical psychologists foster access (or not) to psychological care. More specifically, it interrogates how psychologists manage, and make decisions around, patient referrals. Following a referral, psychologists must resolve an uncertain situation: should they accept a referral and continue with an assessment? Thereafter, they must decide whether a patient is suitable for their service – and for therapy more generally. Certainty is synthesised against a backdrop of sometimes powerful pressures to meet service targets. Taking cues from medical sociology and science and technology studies (STS), this article interrogates some of the uncertainties around access to psychological therapy, and how decisions made by clinical psychologists involve negotiations of patient, service and professional ontologies. To do so, it draws on interviews with 40 psychologists across England and Scotland. The paper spotlights a professional group that is often absent from or only dimly lit within sociological observation and analysis: clinical psychology. Through attending to the discourses of psychologists, I extend conversations about uncertainty through a distinctive case study.

## Introduction

Across the UK, efforts have been made to enhance citizen access to psychological therapy. These include the prominent English Improving Access to Psychological Therapies (IAPT) programme, but IAPT is by no means alone (Pickersgill 2019, Pilgrim and Carey 2012). Rather, a variety of initiatives have been deployed to increase the numbers of people who are able to obtain psychological care. These changes have occurred as reports also suggest that beyond IAPT and certain other services, funding changes for mental health care more broadly have resulted in cost‐cutting measures, created workforce challenges and comprised the quality of patient care (as discussed in Gilburt 2015). Waiting list targets have become a key tool to drive forward efficiency, accountability and the enhancement of access (cf. Sheard 2018) (e.g. the ‘HEAT’ targets in Scotland; Greer *et al*. 2016). Clinical psychologists are providers of and gatekeepers for therapy, and often subject to the demands of targets and the pressures that they introduce.

Despite their importance within the NHS, specific sociological attention to clinical psychologists has been rare. This article is concerned with the practices of clinical psychologists in fostering access (or not) to psychological care. It interrogates how psychologists manage, and make decisions around, patient referrals. Following a referral, psychologists must resolve an uncertain situation: should they accept a referral and continue with an assessment? Thereafter, they must decide whether a (potential) patient is suitable for their service – and, indeed, for therapy more generally. These decision‐making practices involve a form of morally inflected professional judgement (Styhre 2013) through which certainty is synthesised against a backdrop of sometimes powerful pressures to meet service targets. In navigating uncertainty, psychologists – like other mental health professionals (McEvoy and Richards 2007) – have to juggle service demands and pressures, patient requests and concerns, and their personal ethical and epistemic frameworks for considering who should receive therapy and why.

This article considers some of the uncertainties around access to psychological therapy, and how (accounts of) decisions made by psychologists involve negotiations of patient, service and professional ontologies. To do so, I draw on interviews with 40 clinical psychologists across England and Scotland. My approach is indebted to research in science and technology studies that has underscored how the nature of things – that is ontologies – are brought into being through discursive and practical work, rather than necessarily and straightforwardly pre‐existing social interaction (e.g. Law 1999, Mol 1999). The article contributes to the sociology of mental health through presenting new empirical material about a professional group that is often absent from or only dimly lit within sociological observation and analysis: clinical psychology. Through examining the discourse of clinical psychologists, I extend conversations about the nature and effects of uncertainty through a distinctive case study.

### Uncertainty work and mental health

Uncertainty can be regarded as an affective state of individuals acting within specific socio‐political and clinico‐epistemic infrastructures. It can also be viewed as a constitutive characteristic of biomedical and healthcare practice in which clinicians and researchers variously both live with and set about clarifying different kinds of ambiguity (Calnan 1984, Fox 1980, Timmermans and Angell 2001). As Gerrity *et al*. (1992), for instance, have shown, uncertainty is not necessarily something that practitioners refrain from acknowledging. Still, it is often – but not exclusively – “worked upon in medical settings to effect its elimination” (Ross 2017: 90), with certainty “a moral ideal to be achieved” (Adamson 1997: 135). In conditions of uncertainty, expert knowledge can act as a key resource within (accounts of) decision‐making (Sulik 2009), serving to reinforce practitioner dominance within clinician‐patient interactions (Broom and Woodward 1996).

The practices of clinicians might not be directly or primarily experienced and self‐categorised as being carried out in a state of ‘uncertainty’, while nevertheless the tasks being undertaken are precisely aimed at making certain an undefined plan of action. Such ‘uncertainty work’ (Moreira *et al*. 2009, Pickersgill 2011) can seek to clarify, for example, epistemological and normative ambiguities: What do we know and how should we act? And what futures will be brought about if we do? Uncertainty is thus productive (Moreira *et al*. 2009, Reed *et al*. 2016): working towards a resolution of ambiguity can involve the imagining of different futures and the animation of affects and action to realise or avoid them. While uncertainty might prompt epistemic development or animate sociality, this might still not result in a resolution regarded as satisfactory by those participating in uncertainty work (e.g. clinicians) – or subject to its effects (e.g. patients).

Within the clinical practices of psychiatry, everyday uncertainties are salient for “psychiatrists and patients [who] need to work their way through these in diagnosis and treatment decisions” (Hautamäki 2018: 45; see also Rafalovich 2005). Similar issues are at stake in the realm of psychology, where practitioners must decide what patients to work with and to what ends. This can be tricky terrain for psychologists, given the indeterminacy of therapy: what should be done about a referred patient is not clear in part because what will happen as a consequence of an intervention cannot be pre‐known (cf. Hollin 2017). Decision‐making around referrals can involve both gendered and raced assumptions (Kugelmass 2016), besides (as this article will go on to illustrate) the navigation of less obviously charged matters such as particular framings of a service and of the expertise of therapists that nevertheless can also result in potentially iniquitous forms of professional action.

In this article, I analyse psychologists’ accounts of how they resolve uncertainties about what to do with patients following processes of referral and assessment, and argue that decision‐making requires the negotiation of three kinds of ontologies. These are as follows: the nature of a potential patient, the nature of the service in which they work and the nature of their profession.

## Methods and approach

This article draws from a Wellcome Trust‐funded study of how clinical psychologists negotiate access to therapy and appraise the epistemic and normative dimensions of therapeutic work. This included qualitative interviews conducted with clinical psychologists in a range of NHS Trusts (England) and Health Boards (Scotland). Following ethics approval and prior to the recruitment of individual psychologists, an email was sent to the Heads of Psychology (or similar) in various Trusts/Health Boards. This was first, to ask for permission to interview staff, and second, to disseminate the study details to colleagues if this was granted. Heads of the included Health Boards/Trusts were actively supportive of the study, and respondents (hereafter signified as U#P#, R#P#) were largely enthusiastic about being interviewed. Recruitment challenges thus related primarily to the difficulties of scheduling an appointment for me to visit during the psychologists’ hectic working weeks. Only one Head of Psychology rejected a request for inclusion of their department within the study

In total, 40 interviews were carried out. The interviewees were sampled from four ‘urban’ and four ‘rural’ locations. They were located in diverse services that ranged in terms of size and remit. The respondents varied in seniority, from recently qualified clinicians to very experienced consultants. The interviews were semi‐structured and covered matters such as referral processes, discharges, therapeutic orientation, and concerns and challenges faced in clinical practice. Interviews were always undertaken at the office of the respondents. They generally lasted around an hour. All interviews were digitally recorded and professionally transcribed.

Upon repeated engagement with the data, and through conversations with psychologists during subsequent research, it became clear that while the question of how someone came to access therapy rested on a range of factors, three domains were central. These were the nature of the patient, the nature of services and the nature of psychology as a form of professional practice. Through the process of making certain whether someone should be admitted for therapy, these three ontologies appeared to be negotiated through talk and practice.

To better comprehend those negotiations, I more closely interrogated a subset of 20 transcripts. It soon became clear that while some of the mechanics of uncertainty work differed with context, the overarching constitutive dynamics were apparent across the 20 interviews. I then revisited the remainder of the corpus, finding similarly. To further develop and substantiate my analysis, I specifically coded the 40 transcripts for instances when referrals were discussed. I also appraised parts of the interviews where assessments and readiness for therapy formed part of the discussion, when decoupled from talk about referrals.

In what follows, I articulate the (overlapping) negotiations of patient, service and professional ontologies that played out in the talk and actions of my respondents. First, though, I summarise how processes of referral and psychological assessment play out at a general level. Within my analysis I respond critically to my interviewees’ accounts, while also taking seriously the notion of psychological expertise, and their expressions of frustration – and sometime despair – that access to therapy was not more widely possible.

## Results

### The process of referral

Before seeing a clinical psychologist, most people have to be referred by another health professional; for example a GP. This could be via a letter to a specific psychologist or to a service team. This team might be comprised either primarily of clinical psychologists, or include a range of practitioners; for example community psychiatric nurses, social workers and occupational therapists. R3P1, who worked in a multidisciplinary team, described how “we have a team meeting every Wednesday morning, where we look through the new referrals, and then decide who is going to go and do the assessment.” In multidisciplinary teams, decisions must be made not only about whether to accept a referral but also whether the patient should see a psychologist specifically.

Services vary in which parts of the NHS they might accept a referral, and who from. Services admitting, for instance, “people with *serious* mental health difficulties, or quite *complex* difficulties” (R1P2) might largely take referrals from a Community Mental Health Team (CMHT).^1^ Referrals for people experiencing distress relating to a physical complaint (e.g. cancer) could be from a nurse. In such instances, they might speak to a psychologist prior to a referral to get a sense of fit for service; as R3P2 told me, a cancer nurse might “ring me up or email me about someone, and we'd have a discussion with them.” Hence, and as will be unpacked later, uncertainty work around referrals can take place prior to a referral being formally made.

Referrals can also come from outwith the NHS; for U1P4's child and adolescent mental health service (CAMHS), for instance, these could come from schools and social work. Rarely, “people can refer themselves” (R4P1). U4P1 described their hard work at seeking to grow self‐referral: “We market materials in different languages, you know, bus stops, on stations, erm newspapers, we leaflet every household, we publicise ourselves in the newspaper or any magazine that might be going out.” However, even when a psychologist “would be happy to accept self‐referral” (R2P4), this was uncommon and some of my interviewees were sceptical about it. U2P1, for instance, said that they “would be in danger of being overwhelmed,” while R4P2 noted that “the danger is you get a lot of worried well people coming forward.”

What happens after a referral depends sometimes on its urgency, but also on the nature of the service. In some cases, a single person will screen all referrals, refusing and accepting different patients, and taking some to a team meeting for further consideration. For other services, “*every* referral has to be *considered* at a team meeting before they get allocated” (R2P2). Next, I turn briefly to consider what generally happens if a patient is considered potentially appropriate for the service to which they have been referred.

### The process of assessment

Following a successful referral, (potential) patients will commonly be ‘assessed’ by a service team member. If it is a multidisciplinary service, assessment will not necessarily be a clinical psychologist. Assessment seeks to ascertain the nature of the presenting problem and whether psychological therapy (and that particular service) is the best means of addressing it. The time taken to see a patient for an assessment varies, reflecting (e.g.) the service size, location and focus. R1P1, who worked in an early intervention service for psychosis, told me: “What we try to do as a team is er get hold of people, preferably on the same day that we get a referral at least by phone and arrange to see them within a week.” Other patients, however, might wait far longer. Time from referral to assessment was also shaped by varied waiting list targets, which differed between and to an extent within England and Scotland. R3P4, for example, noted how targets drove “how quickly you have to respond to those referrals.”

Assessment is sometimes framed as the beginning of therapeutic work itself. This might be in terms of building an alliance and collaboration with a patient that will provide a good basis for therapy, and it could entail the recommendation of psycho‐educational resources through which a patient can “skill themselves up and inform themselves” (R2P4) about therapy. Some psychologists described the lines between ‘assessment’ and ‘treatment’ as blurrier still; R3P3, for instance, said: “To be honest, I struggle to distinguish assessment from treatment.” For them, “as soon as you start hearing somebody's story and you start listening to somebody's story you're giving them space to do something that can create change.”

Yet, sharp lines were drawn by other interviewees: R3P4 very explicitly accounted for assessment and treatment as distinct, as part of a longer answer to a question about how they preferred to manage waiting lists. They reflected regretfully how they might say to a patient at the point of assessment that “unfortunately this isn't, this isn't us beginning therapy,” and that they will “be waiting for possibly two years” for treatment.

The process of assessment also enables the assessing clinician to form an understanding of the suitability (sometimes described as ‘readiness’) of the patient for psychological therapy. As will be illustrated below, appraising suitability requires the resolution of uncertainty: will the patient engage sufficiently with the therapeutic process such that sufficient gains are made which justify seeing them over and above another on a waiting list? Are they the kind of person that the service should be seeing? An assessment thus involves a construction not solely of the presenting issue, but also of the person being assessed.

### Negotiating patient ontologies

Towards the end of one my interview with R4P2, I asked what complaints would be made “when psychologists get together.” The answer: “probably the workload, number of referrals that come in.” This refrain was common. Waiting lists were often long, with associated targets something that “dominates my daily life, my nightly life” (U1P3), and decisions about prioritisation consequently challenging. R3P4's comments illustrate how uncertainties around this can be rendered explicit and subject to reflexive work:

We try to think about it from a perspective of, ‘These are our clients, aren't they? These are our patients and we have all these needs, don't we? What's the best way to use our time really? What do you think?’ And to try and think about that. So sometimes generally people just wait chronologically on that list, but sometimes people will be prioritised. Erm and it feels to *me*, erm that as the lead, part of my role is to, probably, erm be somebody that people look to, to say, ‘What do you think, [R3P4]? Should we be prioritising this person?’ That always feels to me like a really important decision, because on the one hand it's easy to sit down with somebody in a room and think, ‘Oh, bless, you know, really damaged, really ready to work, what I wouldn't give to start doing therapy with this person right now’. But they're the person that you're in a room with, whereas the other people on the list might just be names. They might just be names because you saw them six months ago and you've forgotten them. They might be names because your colleague saw them and you didn't make that personal connection with them. Erm, so making that decision to prioritise somebody, we try to think of that, well, I think of it as an *incredibly* important decision

The problems of workload and waiting lists were compounded when referrals were framed as ‘inappropriate’. As U1P7 described, “sometimes you'll get referrals and you'll think, ‘that's definitely not for us’.” A referral might be considered inappropriate if someone was drinking alcohol or consuming illegal pharmaceuticals in a fashion that psychologists considered excessive and in ways regarded as contraindicative to therapeutic success. Consequently, substance (ab)use was not an uncommon exclusion criterion for a service; for example by R2P2, who noted that if someone had not “stopped” drinking they would be sent “back to another service.”

R2P1, who worked with children, described an inappropriate referral as follows:

A referral that doesn't meet the complexity for a specialist to a free mental health service but there are lots of other services out there. There's social work for, I mean there are referrals that still come to us where it turns out children are just in an abusive situation and they are presenting with mental health problems, erm but we're not the first line agency.

U3P1 reflected that they might not accept a referral into their service as a consequence of ‘social’ issues:

if people have got a lot of kind of *social* type difficulties, they've got housing difficulties, no fixed abode, that kind of issue, we would tend to want them to be in a position where they've dealt with the social issues before they come and try and look at the psychological issues.

Key, then, to the uncertainty work of determining whether to accept a referral was a requirement to construct an account of a patient, limited not just to their need for treatment. This was often initially undertaken in the absence of the patient themselves, and made actionable through the description of the patient within referrers’ letters, any additional locally available information, and perhaps an informal conversation with the referrer. R4P3 reflected:

it would be easier if things were much more clear cut. But I just don't think, I think you've got to accept there's always going to be a bit of occasional grey areas somewhere. So, sometimes, I phone a GP and ask for a bit more information about a referral, to try and help make that decision as to whether the referral is appropriate

Such conversations could place uncertainty work upstream of the actual referral. As R4P3 said:

if we're referring within the mental health directorate, so that's referrals from other psychiatrists, or the community mental health team, we're supposed to sort of really, instead of just sending in referral, have a conversation with someone before we do that. So we don't end up knocking referrals back

Of course, practices vary. For instance, R4P1 said they would undertake an assessment even in the case of an inappropriate referral:

Even if somebody's quite *extreme*, erm you know, maybe there are issues with alcohol, there might be issues with suicidality, erm I think there's still a duty of *care* to do an assessment of that *person* rather than saying, ‘oh well I can't see them’. I would do an assessment and then link up with whoever I thought more appropriate agencies, or try to with the person's permission as well!

However, they also stated that they were sent inappropriate referrals only “*Very very rarely*.” Hence, the extent to which the ontology of a patient is negotiated at a distance seems, to an extent, to be a function of a wider workload that itself relates to how the service is construed both by the psychologists working in it and by those referring to it.

R2P4 also emphasised the importance of meeting with all patients who pass an initial referral screening in order “to determine if psychology's for *them*.” This determination is a key point of ontological negotiation: it is where a psychologist makes certain that the patient is the kind of person who not only fits with the service but will benefit from therapy. Such a framing of assessment implicitly figures psychologists as having specific expertise not only in the administration of psychological therapy, but also in ascertaining its likely success. This notion of the expert psychologist will be explored more fully in the final empirical section.

‘Readiness for therapy’ was a salient dimension determining if psychological therapy is ‘for’ a potential patient, and psychologists strongly emphasise its import. Ascertaining ‘readiness’ involves the casting of a potential patient as being suitably ‘psychologically minded’, which involves disciplining clinician and service users’ understandings of the latter in particular ways and sometimes through tools like the Psychological Mindedness Scale (Shill and Lumley 2002) (used by R1P2, for instance). Psychological mindedness was largely framed as a means of reflection upon and insight into the self in ways that aligned with credentialed psychological expertise. Epistemic authority for ascertaining this can be delegated – to an extent – to technical instruments, such as the aforementioned Scale. However, not all psychologists used this kind of tool and instead deployed clinical judgement to construct and perceive psychological mindedness within a patient. The uncertainty work of clarifying psychological mindedness thus reaffirms the expertise of psychologists in ways that do not necessarily afford benefit to particular patients, since the construction of its absence can preclude therapy.

One senior psychologist – R2P2 – spoke of the importance of psychological mindedness, while simultaneously reflecting on its lack of conceptual clarity: “I don't think anyone's come up with the definitive idea on that.” R2P2 described how they would often be asked to do assessments for “difficult” cases, with a particular view of assessing whether someone would benefit from psychological therapy. I asked, “how do you make those calls?”; the answer began as follows:

We would look at, erm, what, what, what they're being referred to us as, first of all, and we're trying to move towards when the referral comes in there's a triage system and someone is triaged as suitable erm or not, at the first point of call. So are they keen to be seen? Is it *their* choice to be seen by us or are they being pushed into it? Will they *work* in therapy, and will they attend sessions? Erm, then we also look at what therapies are available for them. And our biggest problem has been junior doctors getting rid of mixed bag personality disorders, who don't want to work [at therapy], who've been in the service for years and actually there is no known evidence‐based therapy to work with them. And they're the ones that everyone thinks, ‘oh, I can't bear seeing that person again’, and sometimes we have to be fairly brutal and say, ‘well, what *can* we do?’

R2P2's concluding question relates to the professional skill of psychologists in appraising a patient's capacity to benefit from therapy. Given other sociological scholarship on mental health (e.g. Kugelmass 2016), there are reasons to assume that the synthesis of certainty in this respect will not be free from bias. As we will see in the final empirical section, navigating the question of whether to work with a patient deemed unable to benefit from therapy contributes to shaping psychologists’ professional ontologies.

In summary, referral and assessment generally entail the need to construct a patient as being like ‘X’, where ‘X’ is a multifaceted biopsychosocial matrix. ‘X’ might, for instance, encompass: (a) patient demographic categories like age and ethnicity; (b) psychological identifiers such as the nature of the presenting issue and readiness/motivation for therapy; and, (c) social and behavioural indicators including employment status, and the existence of networks of support beyond the NHS. Importantly, ‘X’ might also be defined in the negative; as in, ‘not like A, B or C’ (e.g. not a regular user of illegal substances). While patient ontologies were often concretised in person, through the accounts above, we can see that certitude about the in/appropriateness of a referral can also be synthesised in the absence of a patient. In these instances, the inscriptions of a patient found within the text and talk of other professionals are framed as more valuable in limited‐capacity services than time spent assessing a patient face‐to‐face. (Pre‐)Assessment in the absence of patients can thus act as a kind of implicit or even explicit healthcare rationing. A construction of someone as like ‘X’ can be later substantiated, or further clarified, through actual discussion with that person. Aside from the dimension of readiness for therapy, the centrality of certifying whether a patient was like ‘X’ is to ensure compatibility with an ostensibly pre‐defined service ontology, canonising acceptance or denial of a referral as legitimate. However, as we will see, service ontologies are also negotiable.

### Negotiating service ontologies

I was occasionally informed that the establishment of IAPT had resulted in disinvestment in other services and/or increasing waiting lists. As U2P1 put it: “commissioners feel that they're already funding services to take care of patients” needing psychological therapy “but then the services that are being funded don't really feel that they have the *capacity*, or the skills” for particular patients. U3P1 asserted that “we're getting more referrals that IAPT won't see” which “increases the demand on our service, whereas actual resources aren't getting any bigger.” Some psychologists, like R1P4, were concerned that there was “such a gap in service between primary and secondary mental health care that lots of people are falling in that.” Psychologists often told me that waiting lists placed them under “a lot of pressure” (R2P2), shaping decisions about referrals. Partly to manage demand, some services had “very detailed referral criteria” (U1P5) and inclusion/exclusion criteria for what kinds of patients they should see. While some psychologists are critical of these – “I *hate* services that are precious” (U2P2) – they are nonetheless common.

The impact of the wider mental health and social care ecosystem on the nature of a particular service was foregrounded by one interviewee (R1P4) through an example of being referred a patient ‐ such as someone with a head injury ‐ on the basis that the referrer does not “know how or what to do with that person.” R1P4 claimed that “if there were local [third‐sector] services for them,” then “they would be doing those things in the community.” Accordingly, a (perceived) absence of wider health and social services enjoins psychologists into making decisions about the nature of patients and their service.

R1P1 described the flexible interpretation of a service remit in order to accommodate patients judged to be most in need as “a kind of *stretching* of the services.” In a resonant account, R1P3 discussed how they managed a service focussing on “the severe and complex part of, of psychology, related to adults.” Sometimes, though, patients were accepted who belonged to “a group who are, if you like, severe and not complex.” This was, as R1P3 admitted, “probably quite a small number.” Still, it is clear that some psychologists had the agency to reconfigure the boundaries of their service to on occasion accept patients who they perhaps technically should not. This raises questions about equity of access in relation to potential service‐users who are not accepted following a referral.

Less commonly, some interviewees were explicit about the boundaries of their service, reporting a lack of facility to stretch it. R4P3, for instance, underscored how the presence rather than absence of other services impacted how they saw their own service and the patients referred to it. They indicated how the existence of other options for patients enabled them to reify service boundaries. In a discussion about referral processes, they simultaneously constructed both limits for their service and a kind of patient who would not necessarily benefit from it, and validated these constructions through noting that exclusion could feasibly be beneficial:

maybe [a referred patient is] not even appropriate for this service, it might be a case of writing back to the GP and suggesting that the problem seems to be mainly about relationship issues, and they don't seem too severe, maybe they could benefit from, perhaps, from being sooner, from Relationship Scotland, for example. You know, there's agencies that we might think are more suitable.

Resource restrictions and allocations were also sometimes deployed within the interviews as devices through which to negotiate and legitimate service boundaries. Limits to capacity were alluded to by R2P2, for example, while discussing assessment:

It may not be that psychological *therapy's* needed, it's maybe that they need support and that's what we're *very* clear about, the service doesn't offer support. We *have* to do psychological therapy. We're a small service offering, sort of, a specialist component to the mental health unit and *that's* what we really have to do. With no workforce that's important that we stick to the psychological therapies

R4P3 similarly configured an identity for their service in relation to their funds that were available to support its operations:

we had this kind of awkwardness of referrals come in, and it's to do with stress around chronic pain. We've had to be really strict with that, and I mean, that's felt really difficult, because obviously people are struggling. But it's been health related, we've had to sort of explain to the referrer that we can't take them because there's no funding for a post.

Psychologists working in services where they managed the psychological issues relating to physical ill‐health generally appeared to have less difficulty in undertaking the uncertainty work of clarifying service ontologies. These largely related to the physical issue a service was funded to work with. When I asked R3P2 whether there would “ever be instances where you just, the referral just isn't right for the service,” they replied:

Yeah, yeah. Um and I think there are some referrers, and I suppose in our referral criteria, it's, kind of, that the psychological difficulty's meant to come about as a facet of their physical health problem. So, and we do get some people referred who've had a severe injury or a mental health problem, and then have got diabetes, and so we get sent them. And so we do try to pass those back to kind of the mental health teams. Or if it's something that can be managed by primary care mental health, if it's, kind of, panic attacks, even if it is about panic attacks during treatment, then we might, you know, see if they could, they could offer some input. So we do do signposting at that time.

However, even for ostensibly closely characterised services aimed at addressing the psychological dimensions of physical ill‐health, stretching could still occur when, for instance, “clinical judgement” (U1P2) suggested this was necessary. As R3P2 continued:

there's also issues about unmet need, I suppose, and things like, um, pain services, um. There isn't any, um, service at the moment for kind of a pain management programme where I'm based, and so I get sent quite a lot of referrals for that. And that's not always been a consistent thing. Sometimes I have kind of had a couple of people on my caseload who are from that route, but as our service has developed and as the lead from my service is trying to think about developing a, a pain service, but asking for more money for that, we've kind of said we're not going to take those referrals but we're going to log how many that come through to us

These comments demonstrate that the stretchability of a service can itself shift and change over time. Again, this is accounted for as a consequence of the wider ecology of services. In the case of R3P2, a decision was reflexively made to exclude patients to galvanise support to develop a new service. Hence, denial of access can be presented as a rational course of action that reflects the clinical and economic realpolitik of the NHS.

To recap, formalised service boundaries were often put in place to manage waiting lists; yet, psychologists could sometimes exercise their autonomy to renegotiate inclusion/exclusion criteria and accept particular patients. Reconfiguration of a service ontology might be in response to wider resource challenges and the risks of patients falling between the cracks of different services. Resources were similarly introduced by respondents to account for why they might not take on particular patients, ossifying service ontologies. Likewise, the presence rather than absence of alternative services could be invoked to concretise service boundaries. The uncertainty work involved in negotiating service ontologies, then, was in part a function of the wider economic and organisational context within which psychologists were embedded. At the same time, clinicians also exercised individual agency in how they engaged with this context in the resolution of uncertainty moments through implicit and sometimes explicit judgements about who is most in need of access to psychological care. Sometimes, this will mean accepting people for therapy even though they did not quite fit the formal service remit. Others who are referred for different reasons or whose presenting issues are constructed differently by a clinician will not, however, be accepted into a service. Service ontologies are consequently negotiable, yet not infinitely so ‐ increasing the uncertainty work of clinicians, further complicating the algebra of healthcare rationing, and (dis)advantaging some patients over others.

### Negotiating professional ontologies

Through talk of referrals and assessments, my interviewees tended to negotiate their own ontologies as professionals as well as those of patients and services. During the fieldwork, two key moral and epistemic constructions commonly emerged – the good psychologist and the expert psychologist. Within these, psychologists as individuals and psychologists as a professional group can be regarded as synecdochical.

Above, I described the uncertainty work involved in successfully accepting a referral often entails a requirement for the psychologist to construct the potential patient as being compatible with a given psychological service. I also showed how the nature of a service can sometimes be reflexively reframed to accommodate a particular (kind of) patient. This can even include someone framed as unready for therapy. R1P1 reflected on this as follows:

locally there hasn't been a high percentage of people who are being told that they're, they're not ready for a service. Actually I suspect there might be a lot of people who are *not* ready actually, but who are offered a service. I think partly that's been produced by the fact that often they're not in touch with anything else, so there's pressure on the person assessing to say well we need to provide‐they clearly need something.

This account appears to be framed critically: R1P1 seemed to imply psychologists should not take on patients unready for therapy. However, they also validated such decisions through underscoring mitigating factors; that is a “pressure” to admit someone to a service in order to deliver at least some kind of care. Hence, psychologists are also presented as professionals who do what they can to help in difficult circumstances: they are good people.

Notions of readiness and psychological mindedness were leveraged in some interviews in discussions that presented exclusion from therapy as a form of care, and which therefore preserved the clinical and moral identity of a psychologist as ‘good’. R1P2 explained that it was important to ascertain that someone was ready for therapy before accepting a referral, reflecting:

it's kind of er making sure that person's actually ready and motivated for treatment, so they don't come in er and come out too quickly in terms of that they're not ready, and they've often…kind of research of that, you know, are treatment failures at this stage as often people go through an awful lot of er interviews and assessments, and they'll end up not being able to manage the therapy and then dropping out and then feeling quite a failure about it.

Yet, through the course of the interview it appeared that R1P2's default position was in fact to accept someone into therapy. They described how they would undertake psycho‐educational activities with a patient to increase “psychological mindedness so that they really are open and ready” for therapy. Consequently, R1P2's talk stabilises them as a good psychologist who exercises evidence‐based clinical judgment (Timmermans and Angell 2001) through (i) describing work with patients to actively assemble them as subjects amenable to therapy, (ii) implying that not accepting someone for therapy is a form of care, since it would prevent them “feeling quite a failure” if not ready for a psychological intervention, and (iii) alluding to research that grants epistemic legitimacy to their account. Similar sentiments were expressed by U1P9:

if you take on someone at the wrong time the chances are they might have a negative experience of therapy, so they'll have an experience where therapy hasn't worked and then that makes them *much* less likely to come forward again, so you've got to be very mindful of that.

An account of readiness for therapy was not always essential in constructing a psychologist as ‘good’ when not accepting someone for therapy. U4P2, for instance, noted that “if the person doesn't really sit with us” then “I think it's our responsibility to really help the person to be signposted” to another service. Through underscoring the assistance that would be provided to a person in absence of psychological treatment, U4P2 (and a number of other interviewees) accounted for themselves as a caring practitioner even while describing a context in which they would refuse access to their service. Similarly, U1P1 reflected on the different ways in which they upheld the exclusion criteria for their service while also asserting: “I don't like the fact that services are too stringent […] because I think that is actually just a way of excluding people.”

Closely relating to the good psychologist was the figure of the expert psychologist, who was, for instance, skilled in making judgements about readiness in the first place. Issues of expertise were commonly raised in discussions about access to services, with respondents sometimes foregrounding the different kinds of expertise brought to bear in practices such as multidisciplinary assessment (or discharge) meetings, as well as in treatment itself. Regarding assessment, U4P1 reflected that some CBT therapists are “not able to really *fully* assess or formulate and think about engagement” whereas clinical psychologists “are able to do that. And I think that skill is quite unique, which really enables engagement.” In relation to treatment, R4P3 described how in their service “the slightly, I suppose, less complex cases, would go with the CBT therapist, and the clinical psychologist would take the more complex ones.” Hence, we can see that in times of straitened resource, where psychologists are an expensive professional group and waiting lists threaten to grow and grow, boundaries of expertise can come to be drawn and reified.

The construction of expertise was also enabled through a discourse of scarcity; in R4P3's words, psychology was a “really limited resource.” One dilemma relating to this was reflected on by R1P3, who talked about the challenges of undertaking some kind of ‘stop‐gap’ work with patients on long waiting lists; for example “bibliotherapy with occasional meetings.” However, a consequent “conundrum” is that “*every* time you do that you take aware from, you're using the same resource that's been used to do the end‐point therapy.” R1P3 did not think “psychologists should be doing that, actually” – “someone else should be doing it!” Clinical psychologists can thus configure themselves as having highly specialist and uniquely important skills subject to implicit or even deliberate rationing.

Expertise and a discourse of scarcity also figured in R1P3's account and that of some other senior psychologists when discussing who they might themselves see for assessments and/or therapy (e.g. R2P2, R3P4). Since the capacity of clinicians in high‐level management roles to work with patients directly was limited, they or their team might ‘pick’ particular patients who would be deemed to benefit from either their particular constellation of expertise, and/or depth of experience. As R1P3 described, their team “would ask me to see particular people who they thought it would be a good idea if I saw them.” R1P3 went on to frame themselves as follows: “[my team] know that I'm a reasonably flexible, integrative sort of therapist, and that I can form working alliances with, with difficult people.” For senior psychologists, then, the uncertainty work undertaken by other psychologists regarding not just whether a patient should be seen, but also who should see them, enjoined a reflexive negotiation of their own identity in relation to skillset, experience and indeed disposition. Of course, central to this was also a negotiation of patient ontologies prior to therapy itself: were they the sort of person who required the costly resource of senior staff time; for example, were they sufficiently “complex” (R3P4)?

This section has illustrated how the ontologies of both the good psychologist and the expert psychologist come into play within practices of referral and assessment, and the work of making certain that a particular patient should be seen for therapy. These ontologies are also a function of the wider clinical and political circumstances within which psychologists are situated. For instance, claims to (greater) expertise are strategically useful in an economically orientated mental health policy context that positions other professionals – for example CBT therapists – as the answer to the problem of restricted access to services. Constructions of the good psychologist also legitimate difficult decisions made by clinicians when not taking on a person for therapy, or when contravening professional and epistemic norms in order to do so. Finally, the realisation of professional ontologies and the praxis of uncertainty work are reciprocally constituted: it is in part through ascertaining whether to take on a patient for therapy that professional ontologies emerge and are validated and sustained, shaping future engagements with potential service users.

## Conclusion

Decision‐making about who to accept for therapy (and how long to continue it) involves, in the first instance, a form of procedural uncertainty: what, practically speaking, should happen next to the patient? This procedural question relates to the matter of therapeutic indeterminacy (cf. Hollin 2017) – there are multiple possible outcomes of therapy, including limited patient engagement leading to null or even negative effects. The work of responding to procedural uncertainty is a form of skilled judgement (Styhre 2013) that entails the negotiation of three key ontologies: the nature of the patient, the nature of the service and the nature of the psychologist as both an individual and as a professional group. In this respect, movement through uncertainty moments is (psycho)socially productive: action, affects and ontologies are realised through the synthesis of certitude (Moreira *et al*. 2009, Reed *et al*. 2016).

The negotiations psychologists make do not merely reflect realities, they help bring them into being (Law 1999, Mol 1999). While patients, professionals and services obviously pre‐exist uncertainty work, ontologies are clearly negotiated and sometimes reframed through this, rather than patterning outcomes in a linear fashion (Moreira *et al*. 2009, Pickersgill 2011). Patients – and, to an extent, clinicians – can be marked deeply, for better or for worse, by the certainties psychologists produce at the point of referral or assessment, perhaps especially if a decision is reached to offer no therapy at all

Even if accepted for therapy, passing through uncertainty moments makes up patients (and their therapists) in particular ways (Hacking 2002); given the intersubjective nature of psychological practice, this has implications for future care. Accordingly, uncertainty work and the negotiation of ontologies that it entails has normative dimensions. These further include the role of bias (Kugelmass 2016): not as some kind of exogenous entity introduced into a dispassionate and objective professional appraisal, but as a constitutive element of clinical experience that shapes and directs the management of uncertainty and therapeutic (in)action. My interview data suggest how psychologists are empowered to make up patients and services, even within limits imposed by wider economic and organisational factors, in a fashion that can stretch or shrink a service remit, and facilitate or deny access to particular patients. An epiphenomenon of this clinical autonomy will, in some cases, inevitably be the reproduction of forms of structural discrimination within the micro‐sociology of the assessment.

Uncertainty work helps to produce psychologists as both expert and good: synthesising certainties enjoins invocations and practices of the resourceful application of credentialed knowledge towards enabling or enhancing the care of patients. What is important to note, though, is that psychologists can produce different modes of care that are moulded by context (Pols 2003, Schwennesen and Koch 2012). For instance, both seeing a patient who is deemed unready for therapy *and* not seeing such a patient can be configured as worthy acts. Making things certain might entail different constellations of praxis, but psychologists appear always to construct themselves as (trying to do) good. During my research, I was constantly struck by the challenges psychologists face in delivering services, and their considerable regard for their patients. Still, if all choices that psychologists might make can be accounted for as the (ethically, clinically) right ones, this leaves patients who are denied therapy little room for contesting professional power and accessing care.

## Note

1 Throughout, italicisation reflects interviewee emphasis.
